# Supplementary figures and images for: In Vitro Anticancer Activity of Mucoadhesive Oral Films Loaded with Usnea barbata (L.) F. H. Wigg Dry Acetone Extract, with Potential Applications in Oral Squamous Cell Carcinoma Complementary Therapy
Source: Antioxidants (Basel). 2022 Sep 28;11(10):1934. doi: 10.3390/antiox11101934 (PMC9598167; doi:10.3390/antiox11101934)

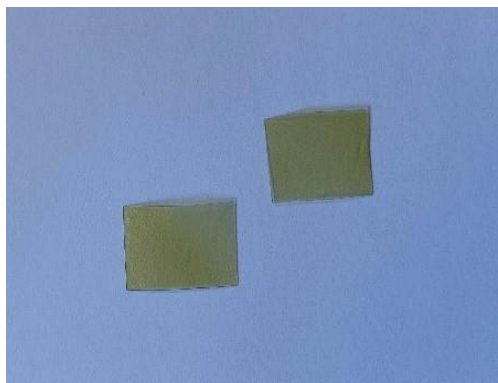

**(a)**

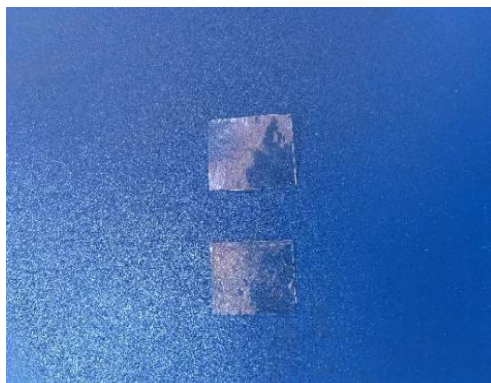

**(b)**

**Figure S1.** Bioadhesive films: (a) Loaded with UBA (F-UBA); (b) References (R).

Supplement: Supplementary file 1 [file antioxidants-11-01934-s001.zip › Supplementary materials Figure S1.pdf]
